# Curiosities of Leech Culture

**Published:** 1862-10

**Authors:** 


Curiosities of Leech Culture.—Many of those who have assiduously cultivated the leech have amassed handsome fortunes, the trade being very remunerative. A prosperous merchant, away in some far district of Poland or Wallachia, will keep some two or three hundred of the inhabitants of his district in full employment, collecting for him, paying them on the best of all plans, according to their labor, viz.: so much a dozen, according to the age and quality of the leeches which they bring to the depot. The animals must be all gathered before the heat of the day sets in, and at once carried home to the capacious reservoirs provided for their reception, where they are at once counted and paid for. Packed in clay or in bags, they are at certain seasons dispatched by fleet conveyances to Marseilles, or direct to Paris, change of horses on the way being insured, when necessary, by liberal payments. The mode of packing the leeches for transport is much the same in most of the breeding districts. Some are placed in boxes—first a layer of moist white clay, then a layer of the little animals, and so on until the chest is full. Some of the merchants pack the leeches in bags as soon as they are taken out of the marshes. Each of these bags contains about sixteen pounds weight, and it is necessary that they should be hung up for a period till the water is all drained out of them, and then the animal rolls itself up into a kind of ball, and lies in a semi-torpid state till it is, perhaps, revived on its journey by a dip into some half-way pond. The boxes or bags containing the leeches are carried in light wagons, divided into necessary compartments. Relays of horses and drivers are always kept in readiness at the various stages of the journey; but, notwithstanding the greatest care may be taken in their transport, immense numbers of the animals are killed. Severe frost or great heat is equally fatal to them.—*Once a Week.*

